# Parole Work in Canada: Tensions in Supervising People Convicted of Sex Crimes

**DOI:** 10.1177/0306624X221144285

**Published:** 2023-01-03

**Authors:** Rosemary Ricciardelli, Micheal Taylor, Katharina Maier, Dale C. Spencer

**Affiliations:** 1 Memorial University of Newfoundland, St. John’s, Canada; 2 University of Winnipeg, MB, Canada; 3 Carleton University, Ottawa, ON, Canada

**Keywords:** institutional/community parole work, sex offender supervision, occupational sex offender programming, parole occupational stress, rehabilitation, desistance

## Abstract

Internationally, parole work is loaded with tensions, particularly when supervising a people convicted of sex crimes (PCSCs) who, due to their criminal history, are stigmatized and occupy the lowest rungs of the status hierarchy in prison and society more broadly. Drawing on analyses of interview data from federal parole officers (*n* = 150) employed by Correctional Service Canada, we interpret their perceptions and feelings about overseeing re-entry preparations and processes for the PCSCs on their caseloads. We unpack the “tensions” imbued in parole officers’ internal reflections and negotiation of complexities in their efforts toward supporting client’s rehabilitation efforts, desistance from crime while negotiating external factors (e.g., the lack of available programming), and being responsible for supervising PCSCs. We highlight facets of occupational stress parole officers experience, finding PCSCs may be more compliant when under supervision but may also require more of a parole officer’s resources, including time and energy. We put forth recommendations for greater empirical nuance concerning parole officer work and their occupational experiences and beliefs about PCSC, particularly as related to parole officer health.

## Introduction

Correctional work, in prison and in the community, is riddled with tensions, including those resulting from job stress. In the current article, job stress refers to the relationship between the Parole Officer (PO) as a *person* versus the demands of their work or occupation as a *job* (Sur & Ng, 2014, p. 81). Specifically, we are concerned with the tension that can arise between the *person* and the *job* when supervising particular criminalized populations—in our case, people convicted of sex crimes (PCSCs). To our knowledge, scholars have only started to unpack tensions—that are often contradictory in nature—which exist in PO occupational work, in general, and PO work that involves responsibility for the successful community reintegration of people labeled criminally as PCSCs, specifically. We refer to *tension* as a state of being tightly stretched—liminal in essence—in terms of moral or emotional conflict, recognizing POs experience stress due to adverse and demanding realities inherent to their occupational work (Brough & Williams, 2007; Owen, 2006; Patterson, 1992; Ricciardelli et al., 2022). In the current exploratory study, we draw on interviews and two focus groups that resulted in a sample of 150 Canadian POs to unpack the tensions around the supervision of PCSCs. The study, however, remains exploratory as the key focus was on POs’ occupational stress injuries rather than their experience supervising PCSCs. The theme of PCSCs arose organically from the data when probed about if they had experiences overseeing PCSCs or when speaking about occupational stress, potentially psychologically traumatic event exposures, or their occupational work more broadly.

Specifically, we seek to understand how rehabilitative factors and orientations can be stymied by correctional systems and societal perceptions, which stand in tension with promoting well-being for PCSCs while navigating the processes deemed necessary for maintaining public safety. This is to say that the interpretations of and dispositions toward PCSCs in Canadian society and the criminal justice system, respectively, hinder POs delivery of rehabilitative services to PCSCs. This is compounded by tensions between PO efforts to maintain a rehabilitative orientation versus the challenges experienced when reading casefiles or the insufficient availability of services and programs for PCSCs on release versus navigating the stigma with which PCSCs live. We ponder these challenges of supervising PCSCs as tension as well, referring here to tensions in, for example, assessments of PCSCs risk classification versus perceived propensity for desistance and how both shape the public safety responsibilities of POs. Moreover, we recognize the impacts of some forms of sex crimes and appreciate that these crimes are not palatable for some POs, which creates a tension around *who a person is* versus *what they did*.

The images of PCSCs in our society evoke persons who cannot be trusted because they violated societal norms, thereby becoming an “outsider” (Becker, 1963). Being publicly villainized, labeled as sick, and deeply discredited as a person, we understand the socially constructed PCSC term to mean someone that has doubly offended sexual mores of civic society that evokes feelings of anger, shame, and disgust and is symbolic of the most hated group of criminalized people (Ricciardelli, 2017, pp. 2–3). This is despite how a great number of offenses fall under the label of sex offender. For example, in Canada an adult convicted of sexually abusing a child receives the same label as an adult that is convicted of purchasing sexual services from an adult sex worker (see Ricciardelli, 2022). PCSCs occupy a large part of the criminological imagination that exemplifies the complex tension between images of suffering and compassion (Carrabine, 2012, p. 69). They represent a popular criminology for ensuing discussion about how to invite philosophical, moral, and ethical perspectives—often tense and polarizing—to create nuance for subtlety challenging presuppositions and distinguishing justice from revenge (Kohm & Greenhill, 2011, p. 212).

Tied to the supervision of PCSCs are tensions, as the PO is responsible for public safety, which entails ensuring clients on their caseloads are well positioned in society and, as such, desist from future crime. Challenging this occupational purpose, remains how PCSCs embody a nuanced understanding and positionality for engendering the past, present, and future. We demonstrate that the supervision of PCSCs can be mentally and emotionally straining for POs, and thereby create occupational and personal tension for POs (Hamin & Hassan, 2012; Kras et al., 2017; Ricciardelli, 2022; Sigler & Mcgraw, 1984; Whitehead & Lindquist, 1985). The supervision of PCSCs takes place within a broader context of tightly stretched resources of correctional personnel, organizations, and communities at large which heightens tensions and stress further (Levenson et al., 2016; Ricciardelli, 2017, p. 91), and contributes to POs’ perceptions around their demanding emotional labor.

Underpinning the tensions of POs working with PCSCs, moreover, is the stress and aversion inherent to “high-effort, low reward” conditions, especially regarding the chronicity of stressful experiences over time (Siegrist, 1996, p. 39). As Raphael et al. (2020, p. 27) explain, a determinant of health in workplace well-being is the effort-reward dimension underlining the importance of *demands* (e.g., time pressures, interruptions, and responsibilities) being aligned with *rewards* (e.g., monetary, esteem, and respect). Over time, aspects of psychosocial work might support and even promote health by balancing the tension between workers’ perceptions of costs and gains in their experiences supervising PCSCs as a job; however, the costs in our society due to interpretations of sex crimes may not balance with gains. Chiefly concerning here is the tension between outcomes of public health and public safety (Raphael, 2016, p. 171; Raphael et al., 2020; Smith & Polanyi, 2016), as emergent in the tensions experienced by POs’ working conditions and their supervision commitments to PCSCs.

### Theory of Stress: Contrasting Personhood and Emotion

Brown (2021) observes that mental and emotional separation is required to distinguish personhood from behavior, particularly when *guilt* is experienced when a person falls short of their own expectations. Guilt results from *doing* something wrong (p. 146), which is contrasted by *shame* (i.e., “intensely painful feeling or experience of believing that we are flawed and therefore unworthy of love, belonging, and connection” (p. 137)). Guilt requires empathy to create adaptive responses for addressing, for example, sexual impropriety and misconduct (p. 147). Shame on the other hand is egocentric and incompatible with empathy because shame is self-involved and draws upon the inward focus of *being* judged by social norms (p. 142).

Behavior and emotional expression are germane to stress and exist despite personalities (Friedman & Booth-Kewley, 1987). While traits^1^ may mediate perceptions of the job-stress relationship, we remain focused on three situational factors: *locus of control, self-efficacy*, and *self-monitoring* (Sur & Ng, 2014). POs possess distinct personalities in their work and nonwork (McKendy et al., 2021, p. 13), and their “dirty work” requires compartmentalization to insulate themselves from adverse demands of the job (Scott-Bottoms, 2020; Spencer et al., 2021). In this way, POs also work to de-compartmentalize the perceptions and social contexts of their supervisees. For instance, *locus of control* refers to the tension between a person’s intrinsic and extrinsic orientations to the situation, wherein the PO represents an extrinsic feature to the PCSCs’ intrinsic perception of self. Likewise, PCSCs represent an extrinsic concern for public safety in the same way we all possess uniquely intrinsic preferences about sexual expression. POs may strive to instill *self-efficacy* by moderating PCSCs’ preferences, fantasies, and behaviors which, if successful, is intrinsically rewarding—for the client who self-actualizes and desists and for the PO who helps facilitate this best-case scenario outcome. By contrast, the tension that comes with “failure” has costs for the extrinsic social world. POs thus *self-monitor* by focusing on worst case scenarios—“nightmares”—regarding their supervisees reoffending and creating more victims while under their supervision. Yet another tension exists in how PCSCs retain secrecy about their true personal feelings that may deviate from social norms.

Following “care ethics,” practices are shaped not by rules and processes but by people and their circumstances in all diversity (Dominey & Canton, 2022). Care ethics start with the ethical assumption that PCSCs are people deserving of the opportunity to live with dignity after being punished (Ricciardelli, 2017, p. 92). The best case scenario (i.e., an outcome of rehabilitative-desistance) requires interrogating the hate directed, rather often, toward PCSCs to appreciate, through a positively imagined future, ways of both seeing and being in the world anew (Watkins et al., 2011, p. 282). Rather than hate, POs supervising PCSCs must engage in meaningful dialog that communicates citizenship and civic responsibility while undertaking client punishment (Duff, 2003, 2021).

The intention of the PO is to morally persuade and help PCSCs desist problematic lifestyles while working toward the goal of rehabilitation and eventually well-being (McNeill, 2006). Neglecting this in PCSCs supervision for POs, inside or outside of prison, contributes to the negative effects of spiraling incivility in carceral workplaces (Andersson & Pearson, 2013). Therefore, the PO job represents a form of *human surveillance* where “regard or attendance to others” occurs by collecting data connectable to the person as a member of a category (Marx, 2015, p. 734). Thus tension remains inherent to correcting PCSCs because of their resistance to the “social sorting” function that is key to help classify and monitor sets of people deemed risky and, sometimes, exclude them from full participation in society (Lyon, 2003; Lyon et al., 2014).

### Risk Assessment: Tensions in Predictive Opinion and Unstructured Nuance

Tension is inherent and paradoxical in supervising PCSCs because sexuality is a large part of self-identity, that is hidden, both intrinsically personal and secretive yet vital to well-being in its expression (Goode, 2009, p. 85). A dilemma emerges with atypical sexualities, such as pedophilia or bestiality, where, for example, a person’s identity is tied intrinsically to a deviant sexual preference, but social norms extrinsically challenge that identity. Problematic sexual interactions include both contact and non-contact offenses, both representing potential public safety and public health threats simultaneously. As Seto (2018, p. 171) explains, managing criminogenic risks and needs of PCSCs is twofold. First, accounting for *atypical sexual interests* including excessive preoccupation, paraphilias, and orientations that deviate social norms is necessary, as is, second, *antisociality*, which includes factors of impulsivity, callousness, and attitudes associated with entitlement. The latter is about PCSCs’ beliefs about themselves—who they are—versus the former, which refers to what they do based on preference. These dimensions are meaningful because atypical sexual interests can be viewed as the motivations for committing sex offenses, whereas antisociality represents factors that facilitate acting on those motivations (Seto, 2018).

Together, these dimensions combined predict the worst-case scenario of sex offending, a concern for POs. For example, a PCSC who is low in empathy and high in psychopathy—*Without Conscience* (Hare, 1999)—as a person who also possess devious interests in vulnerable subjects is a situation where accountability must be prioritized and recidivism discouraged. Imperative remains that contrary to popular opinion, the assumption that most PCSCs will reoffend is incorrect (Seto, 2018, p. 169), and differentiation is needed to understand which cases pose the greatest threat to public health and safety.

### Integrating Models of Client Management

Matching intensive levels of treatment is required for higher risk criminalized people while minimal intervention is necessary for lower risk PCSCs (Andrews et al., 2011; Bonta & Andrews, 2017). While this remains in tension with creating experiences of well-being, it is consistent with the Risk-Need-Responsivity principles of individual assessment and rehabilitation (Bonta & Andrews, 2007, 2017) and with the Good Lives model (Andrews et al., 2011; Ricciardelli, 2018; Ward & Maruna, 2007; Ward & Stewart, 2003), which requires program intensity match an individual’s risk level while being responsive and remaining strength-based.

Criminogenic risk by itself fails to inform *how* criminogenic needs relate to criminal conduct with which case analysis about sex offending “requires paying attention not only to the degree to which a risk factor is present but also to the *meaning* of the risk factor” (Serin et al., 2019, p. 728, emphasis original). While POs practice conducting risk assessments, they are primarily concerned with how quickly a new offense might occur and its severity (Seto, 2018, p. 163). Whereas, motivating individuals to change requires time to concentrate on the dynamic factors (e.g., fantasies, attitudes, orientations etc.) which, unlike static factors, have the capacity to change (Andrews et al., 2011; Bonta & Andrews, 2017). Required here is techniques that move beyond the “reliably sort[ing]” of individuals into categories based on sexual recidivism (Hanson et al., 2007, p. i) to communicating civic responsibility and morally persuade PCSCs through inspiring or modeling how to live a life of dignity (Duff, 2021; McNeill, 2006).

### Intervention as Meaningful and Actuarial

Categories require nuance to understand PCSCs’ lives at the level of interpretation regarding how *they* attach meaning to aspects of their own existence (Ricciardelli, 2017, p. 4). This is the responsivity principle in practice, which requires mutual respect, openness, warmth, and enthusiasm in facilitating a therapeutic alliance through dialog primarily focused on a holistic and comprehensive approach grounded in innate motivation (Ricciardelli, 2018, p. 3). Purely predictive interventions based on aggregation and actuarial assessment fail to account for how abnormal sexualities are uniquely positioned and engendered. Tension exists given the paradox that most self-identified persons with a paraphilia may not ever sex offend evinced by some seeking assessment or treatment through confidential community-based clinics obfuscating actual versus observed rates of recidivism (Seto, 2018, p. 170).

Recidivism is not a robust measure of effectiveness for community correctional work (Butts & Schiraldi, 2018), and stands in stark contrast to the meaningful conception of success offered by processes of desistance and achievement for those leaving prison and to promote personal well-being (Rosenfeld & Grigg, 2022). Each case requires uniqueness, as the *responsivity* principle states, where delivery of services must be in a style and mode that matches that of the individuals’ in reference to cognitive-behavioral and social learning strategies (Andrews et al., 2011; Bonta & Andrews, 2017; Bourgon & Gutierrez, 2012). Of course, all of this takes varying degrees of time which is more than just counting program hours (Sperber & Lowenkamp, 2017); instead applying “dosage” in terms of how and why therapeutic programming relates to the individual, which must be accessible, relevant, and flexible.

Whereas dosage plays a mediating role in the level of service, or amount of treatment, a PCSC may require for initiating meaningful change in the community the transference of Risk, Need, and Responsivity principles is more difficult in prison (Bourgon & Armstrong, 2005). Empirical guidelines about treatment dosage continue to remain elusive and difficult to quantify (Serin et al., 2019), because, qualitative nuance is required to attend to identity through one’s own narrative (Aas, 2004). Creating interventions that are holistic and understand the whole individual requires moving past mere attributes of a person based on “ticked boxes” (Fitzgibbon, 2007). Desistance is not reducible to simplicities by applying the right treatment at the right dosage (Mcneill, 2004), but by creating balance between the tension of “unstructured” professional judgments with “actuarial” risk measures (Seto, 2018, p. 179).

## Methods

Parole work in Canada has received little attention especially compared to research on incarceration and correctional officers. In the federal system, parole officer caseloads are around 30 to 35, workloads are high, and many experience mental health disorder prevalence (Carleton et al., 2020). Given, we have described the data and methods elsewhere (see Ricciardelli et al., 2022), we keep this section relatively brief. In essence, we base our analysis on interviews with 96 participants working as Institutional Parole Officers (IPOs), and 54 participants as Community Parole Officers (CPOs). Whereas IPOs and CPOs have similar labor processes, in Canada the key distinction between the two is that IPOs make recommendations to the Parole Board of Canada regarding the early release of prisoners; CPOs make recommendations on some of the conditions that they deem necessary for the early release of prisoners. Most interviews (*n* = 145; 96.7%) were conducted one-on-one in English. The remainder (*n* = 5; 3.3%) were organized as French-language group interviews, which were translated in real-time. We conducted interviews between August and October 2020, and, due to COVID-19 public health measures, over the phone. The sample included IPOs employed in the eight Canadian provinces in which there are CSC correctional institutions, while the CPO sample drew on participants from seven provinces and all three territories. We summarize participants’ (IPOs and CPOs) basic demographics in Table 1.

**Table 1.** Parole officer demographics.

	Participants (*n*)	Participants (%)
Gender		
Female	114	76
Male	33	22
No answer	3	2
Age		
19–24	3	2
25–34	21	14
35–44	64	42.7
45–54	42	28
55–64	15	10
65–74	2	1.3
No answer	3	2
Race		
Indigenous	2	1.3
Chinese	4	2.7
Black	4	2.7
South Asian	4	2.7
White	128	85.3
No answer	3	2
Racialized other	4	2.7

Interviews were approximately 1 to 2 hours in length and followed a semi-structured interview guide. Interviewees were asked a range of questions, including how they viewed their responsibilities, the challenges and rewards of their work, the emotional impacts of parole work, exposure to vicarious trauma (Burruss et al., 2018; Moulden & Firestone, 2007), and the impacts of COVID-19 (see also Norman & Ricciardelli, 2021). For the current study, we analyzed participant responses to items asking if they had worked with PCSCs and their experiences in so doing. Research assistants transcribed interviews verbatim.

Our approach to coding was open-ended, using a semi-grounded approach, to identify emergent themes (Charmaz, 2014; Glaser & Strauss, 1967, 2017). We developed a preliminary set of codes by independently and sequentially coding five transcripts. Subsequently, the research team coded the remaining transcripts, refining existing codes and creating new ones as they emerged from the interview data. We used QRS NVivo Pro to assist with autocoding and coding data into primary, secondary, and tertiary themes, and employed axial coding to make connections between and further develop these themes (Corbin & Strauss, 2008).

## Results

We structure the results to first unpack dedication by POs holding a rehabilitative orientation to help support PCSCs on their journey toward or when re-entering society. Then we explore the tension around available (i.e., insufficient or non-existent) programming and service provision to further assist POs support PCSCs on their caseloads. Next, we turn to tensions in the ease of supervising PCSCs juxtaposed with the time-consuming nature of some cases, recognizing not all cases are equal in time requirements. We turn then to the tensions in supervision—the conflicting, even contradicting, realities that shape the supervision of PCSCs. Here, we highlight tension in comprehension of criminality, risk assessments, predicting recidivism, and of simply at times being unable to work with someone due to their criminal histories and sexual deviance.

### Maintaining a Rehabilitative Orientation

POs spoke about the tension inherent to maintaining a rehabilitative orientation when dealing with the shame felt by PCSC clients. The shame caused POs to struggle with how to deal with their clients and their criminal past. POs believed embarrassment and shame were a hindrance to creating an open dialog for conversation about risk and rehabilitation. P90, for instance, explains:

There’s so much shame attached to it that they either can’t recognize it or won’t recognize it or they are just too embarrassed to talk about it… So I come to work and if I have to discuss like specifically risk relevant issues with a sex offender, I have to kind of take my, my personal path of my own beliefs and be like okay, ‘I need to get information from this person who doesn’t want to tell me’… So I have to convince somebody that I’m not going to be judgemental. That I’m here to help and to understand and not to berate or shame them. Cause otherwise they’ll just shut down, they won’t tell you anything.

P90s words speak to the tension in desiring to act in a rehabilitative function when trying to navigate their client’s feelings, specifically shame and embarrassment, that may come in the way of building open and trusting relationships. Similar to the findings of Skeem et al. (2007), POs must balance the dual roles of care and control in managing PCSCs. Concomitantly, they struggle to have some PCSCs disclose personal feelings about their criminal history because of guilt and shame. The PO’s concern is that the PCSC is left without support because they fail to disclose and receive the help offered, which hinders the PO’s ability to perform a more rehabilitative function (much like a paucity of preventative supports for PCSCs leads to the paradox that one must first offend before they may receive support). Another interviewee, P36, also spoke about the goal orientation toward successful desistance from crime and success in re-entry that shapes their occupational work. Speaking more specifically about the challenges of supervising and providing support to PCSCs in the prison system, they explain:

it [their offence] doesn’t change how I work with an offender whether it’s a sex offender or a non sex offender. Same thing. We’re working towards a goal. Making changes, making sure they get their programs. Making sure that they do everything they need to do. And then ensuring that they’ve made all the necessary changes and then moving them back out into society… The challenge sometimes with working with sex offenders, I’ve found is more the institutional setting. Sex offenders generally are not particularly liked within the inmate population by other inmates. And so you run into—and sort of managing a sex offender can be difficult at times because they may end up having more issues with other offenders you’ve got to work at trying to find a place where they can be safe and trying to ensure that you know, they, they—that their safety isn’t compromised by other offenders.

As P36’s words speak to, even when a PCSC can open up and accept support, these prisoners are still plagued by the social hate directed at them because of their criminalities, which then impedes on their ability to access necessary supports. PCSCs fall to the lowest rungs on the hierarchy of prisoners (Ricciardelli, 2017; Ricciardelli & Moir, 2013; Schwaebe, 2005), which leaves them susceptible to harm, particularly when in prison. This additional burden, to support client safety, impedes on POs’ capacity for dealing with the underlying challenges tied to supporting the rehabilitation of PCSCs because instead, officers are distracted by managing day-to-day client safety needs. To this end, attesting the rehabilitative orientation that is pronounced among POs to supervising PCSCs, are the words of P46: “I don’t personalize or try [to] judge somebody based on the offence, it’s based on the risk and that’s what I’m focused on.” Similarly, P7 explains that:

I think it takes a certain type to kind of go with the flow at times, and be respectful towards inmates who have done pretty, pretty terrible things. I mean I have heard myself saying: ‘I certainly don’t like what you did, but you’re here as a human being, and we’ll work from there’, especially the sex offenders.

As these POs’ narratives show, POs sought to act in rehabilitative ways and engage in open dialog with PCSCs, which they thought was critical to client rehabilitation. However, they believed their work, and therefore their potential to work on a person in more helpful and rehabilitative ways, was made difficult due to the shame and stigma that is attached to the label of PCSCs, which is both internalized by PCSCs themselves and plays out in interactions among prisoners that, in turn, affects their own work.

### Lack of Services for Sex Offenders

POs expressed concerns about the accessibility and relevance of available programming for PCSCs. Some, like P19, noted with a sigh “I question whether our programming is actually doing anything,” expressing concern about the impacts on the PCSC of the programming. Others expressed concern regarding the lack of programs for certain groups of PCSC, or talked about exceptions to programming qualifications. Here, P90, explained:

One thing I find very, very, very frustrating is um, guys who are only convicted of um, incest because with that everybody—all the excuse me, all the research says that this guys a low, low, low risk because he only offends against his own children and if he doesn’t have his own children then he won’t offend. And with that because if he’s gonna be super low risk like that, we don’t have low intensity programming. We only have moderate and high. So essentially, if a guy is too low on the risk scale, he gets zero treatment.

Sentiments about the lack of available programming were echoed by others, who expressed concerns about persons not qualifying for programming because of the nature of the crime. Others spoke of difficulties in “having to kind of separate them from what they did and still being able to provide them with the services that can help them” (P14). The PO continued explaining that, particularly in the “north … there is no services here for them in that regard, in the regards of sex offences” (P14). The lack of services and programming was frustrating for POs, which is most readily apparent when discussing assessments of PCSCs. Here, to demonstrate, P30 speaks to how, while supervising PCSCs:

I can’t get into his head and nor do I have the time to sit with him every single day or every single week and talk to him about it, so, it’s hard. It’s a challenge to access them fully. I know before we used to have [a] psychologist that would be in. They would do their sex offender programming, there would be a program facilitator and a psychologist. We don’t have that anymore and I think that that unfortunately is a big loss…

P30 alludes to cessation, in their experience, of the shared case management that assist with PCSCs assessment as “a big loss,” continuing to describe how the need to supervise and correctly assess PCSCs is instrumental for the continued support of public safety. P7, also reminds us of this reality, explaining:

It’s very hard to work with, with them. But you know, there’s a job to be done. And at some point, they’re going to be released. So, our job then I think its to prepare them as best we can for that, because you know, they could be living next door to you.

The essence here is that without the necessary programming and services, the onus of responsibility falls onto POs to ensure their clients do not reoffend and instead are supported to safely return to the community. POs, however, also experience external constraints (e.g., high caseloads, lack of time), meaning their time and ability to focus intensely on the supervision of PCSCs is limited.

### Easier to Supervise But Also Time Consuming

Despite the aforementioned challenges, POs believed supervising PCSCs in both the institution and community was eased by PCSCs’ general personality and behavior characteristics (with some caveats). Regarding institutional supervision, P121 explained that “their institutional behavior is often a little bit more positive because they stay quiet,” while P30 notes that “they’re generally compliant. They don’t get involved in institutional problems, they’re not involved in the drugs they [are] generally decently educated. So okay ‘you’re like a superstar inmate, but what about [the] sex deviance part?’” Here, P30 points to the tension between crime and personality—being easier to supervise but sexually deviant in often unforgiveable ways. P54 says:

I have lots of experience with sex offenders. Sometimes they’re easier to manage and sometimes they’re harder to manage. Sex offenders typically and this is not like society cannot wrap their head around this, sex offenders are more manageable than others, like than a property offender or than like a bank robber or B and E guy and that’s really hard for most people of the general public to swallow. I mean our sex offenders are those that are the very most high risk and so these guys are not easy to manage.

P54 points to the tension between the personalities of PCSCs and their sexual deviance, finding PCSCs easier to manage in personality but harder regarding the complexities of their sexual preferences. This tension was also expressed by P24 who works with “quite a few” PCSCs and explained that they are:

more compliant [than other offenders] and with it, so when you’re dealing with them, interacting with them on a day to day basis you’re not going to have issues. But they’re more secretive in terms of what they’re doing and their activities and when they do reoffend it’s they’ve been in the crime cycle for quite awhile and you just didn’t know it.

This fear of what a PCSCs is capable of, is a challenge, but the challenge is often in tension with the fact that, as per P19, “they’re the easiest inmate to work with, super compliant, don’t really get into any trouble in the institution they usually progress through their correctional plan no problem, like take their programs and, and you know that kind of thing.” P5 echoed:

they’re quite polite, not to be generalizing, but yeah I am generalizing [laughing]…Like, they’re quite friendly, they’re pleasant to deal with, but I think most times that they’re not even aware that they’re doing these things, like sometimes I don’t think it’s on purpose.

Although many spoke of PCSCs as being “easier” to supervise due to their comportment and positioning, some also felt that PCSCs whose cases were “high needs” or who failed to take accountability for their actions, were very difficult as well as time consuming cases. P6, talking about the workload accompanying many of such cases, explains:

they don’t take into consideration when you have those high needs cases cause I’m sure they can argue that everyone’s going to have a high needs case whether it’s mental health or high profile or just an icky sex offender or something like that.

In essence, POs speak of how not all cases are equally constituted in workload, thus, a PCSC who requires greater oversight is actually a drain on time and resources—personally and organizationally. Others, echoed the sentiment, explaining how diverse conditions of release can equate to additional hours of oversight, here P14 explains “a few of them would have sex offender registry requirements, some might have restrictions on being in the presence of children, or [can] not be in the presence of children.” Such additional requirement take more time and more vigilance. Such due diligence is key, as P57 disambiguates:

the stress levels, [become] a little higher, [in] the possibility of re-offending, for parole officer and supervisor, that’s pretty much, our worst nightmare is [sexual] reoffending. … if an offender reoffends, shoplifting is a much different story than if it’s a sex offence. But the consequences are so much more serious and, and brings a lot of stress.

Thus, the added stress of the potential for re-offense makes these often more compliant PCSCs more stressful to supervise. Here, P126, puts forth this complex space and the associated burden that makes such cases difficult:

They’re very likeable in personality, very compliant, very kind, but then it’s also like at the back of my mind being like have you been around any children, are you in a relationship, where do you go on your spare time. You’re constantly like ‘yeah, sure you’re incredibly likeable in front of me,’ I’m like, at the same time, ‘you’re convicted of an offence where you ruined someone’s life’.

### The Tensions in Managing Such Cases: Not Being Able to Comprehend Crimes

Tension underpinned nearly all discussion around the supervision of PCSCs. Tensions took different forms, but often were based in lack of comprehension about processes and details surrounding cases. Some participants spoke at length about how the fact that PCSCs who are considered low risk, despite their crimes and the violation their crimes caused, results in their successful application for parole. Here, P118 says:

I mean it depends on the nature of their offense. The frustrating thing is that a lot of guys that you know commit crimes with child pornography and things like that, they’re considered low risk. So they’ll come out on a day parole or something and then you catch them connecting [with] under age kids, catch them with pornography. A lot of times they’re considered low risk. so ‘let’s get them out of jail’. But they come out and they go right back into their offence cycle and that’s just kind of frustrating.

P118 speaks about how low risk of harm does not translate to low risk of recidivism and the frustrations that result from reoffending that, they feel, could have been delayed at least with a longer sentence. P43, shared similarly feelings, expressing that

I just had a sex offender who takes zero responsibility get parole. And so it’s a little bit of a head baffling thing. I understand what, what they’re saying because his offending was in the context of family so they’re under the guise that as long as he’s not in a family relationship with someone with a young child then the risk is being managed.

P43 recognized why the former prisoner received parole, but does not feel their criminality and consequence aligned, which is beyond their control but still difficult to reconcile. Other participants spoke about the tensions around the crimes that resulted in the PCSCs label—and how the label was not entirely representative of the diversity of crimes—grouping people together with a stigmatizing label without deciphering nuances. P64 provides an example of this tension:

I’d had a sex offender—he was in his twenties but he had sex with a 17 year old boy—it was a homosexual relationship but the 17 year old was still under age. It’s a really bad thing to do right because I mean they smoke pot and drank and then he took advantage of him and all this sort of stuff but from my perspective you can get parole for sure…because I’m used to the guys whose taking woman off grabbing them and raping them in the bushes right, so in that way I’m thinking this guy is a good guy. So in some way, outside of this office, it’s hard to explain how I could support that guy to get out on an early release. If I tried to explain that to someone who walk up to on a street ‘how could you possibly support someone who’s in for sexual assault?’

P64 voices the tension in how not all crimes “fit the label” but also in how difficult providing support for PCSCs can be because of the stigmatizing label. Knowing they live with this tension, the officer alludes to the isolation that can come with their occupational responsibilities due to the lack of public understanding around what constitutes, or fails to constitute a sexual offense. Other tensions arose in cases where PCSCs failed to account for their role and their criminal responsibility. Here, P26 explains that:

Some of them, it can be difficult because they don’t see anything wrong with what they’re doing. They really distort what happened and their version is drastically different than that of the victim based on the police report document.

P26 speaks to the lack of accountability and in essence, being torn between two versions of the same incident. In the PO role, there is no judgment—the judge lays the sentence and convicts, thus deems what is now official truth. Thus, they experience the tension between the “official” version of an incident versus that reported by their PCSC. In essence, they are occupationally responsible to view the official version as truth and having a different version is a signal of being “unchanged” and not “remorseful,” which affects both their occupational work but also their ability to understand possibilities for the PCSCs to reengage in similar crimes because they appear as failing to understand and take responsibility for their actions.

Despite the desire to not be judgmental, some POs felt the tension tied to simply not being able to comprehend the nature of PCSCs atypical “sexual deviance” and thus their antisocial tendencies. Officers here spoke of specific cases they could not oversee for this very reasons—some PCSCs’ actions were too disturbing for them to be able to truly provide support. P49 explains:

I remember having a conversation with one guy, and he was only here for a few weeks and he was going somewhere else. And, he said to me, he goes ‘I know I’m disgusting.’ … But that’s so few and far between ‘cause, for the most part, I’m really able to separate it. But every once in a while there’s someone that comes across that you’re like ‘oh, I don’t even know if I can actually deal with this person’.

P49 could not, and ultimately was not obliged, to continue supervising a particular PCSC because of the details of his criminal actions. This created a tension between wanting full occupational responsibilities but also not having the capacities to do so because in providing support for re-entry, they felt they were compromising public safety—a major objective of their work. This was echoed by P1 who told the story of a PCSC on their caseload:

I have an offender on my caseload right now who I worked very hard with the psychologist last year to deal with um but he had sexually assaulted his daughter over the course of eighteen years. He also was taken to bestiality and had sex with horses and dogs and other farm animals and he would force his daughter to have intercourse with horses and dogs and he would. if she wouldn’t do it. he would cut like the paws off of her kittens or cut the heads off her cats in order to get compliance and that case is super twisted obviously. But talking to him what I struggled with is he would talk about horses like normal guys would talk about blondes versus brunettes and he found such a strong attraction to dogs and horses. I couldn’t wrap my head around it… It it was so outlandish and so twisted um I really struggled with his case. And then when I’d interview him he would spend a lot of time talking about avoidance techniques like he would be caught by the officers masturbating to watching a movie called *Seabiscuit*, it was about a racehorse. And he he got off on it, so we would have to discuss techniques to stop watching nature shows or animal shows.

The PO speaks about the disturbing nature of their client’s criminality and the processes they must engage in to help the PCSC refrain from further criminally violating activities. The tension arises because they too could not comprehend the nature of the client’s affiliations and, in consequence, the harm they caused. Similar sentiments were echoed by others, like by P25, who explains that working with PCSCs “bothers me as a human being but it’s ok. It doesn’t scare me.” Others explained that sometimes the disturbing nature of one’s criminal acts are beyond comprehendible or imaginable, thus “not what anybody signs up to do” (P1). Here, P1 elaborates:

Being a parole officer, in college, they never say ‘well, this is what you’re going to be doing’ and in the end it’s like ‘oh this shouldn’t be specifically said like a guy that there’s no hope for he’s doing a forever sentence he’s a dangerous offender.’ Basically well, he’s a lost cause and I only talk to him when I absolutely have to now. And we avoid talking about his deviance cause he’s so screwed up I’m not going to fix it, a program’s not gonna fix it.

P1 speaks to the lack of training and understanding that not everyone, while in prison and prior to their release, is ready for desistance from crime or to be rehabilitated. Of course, this is not the norm, as P9 reiterates: “With those who have some sort of not redeemable features but the willingness to change to even acknowledge that they have crossed the line of society, you hold onto those threads and go ok there’s something I can work with here.”

## Discussion: A Four-Point Axis of Tension in Parole Work

In the current article, we examined POs’ perceptions and feelings toward supervising PCSCs. POs, tasked to supervise PCSCs to reduce potential future harms, also work to understanding PCSCs’ sexual expression, past criminality, and need for rehabilitative interventions to aid in their desistance of crime. For POs, imbued in the supervision of PCSCs are tensions and at times opposing feelings that are difficult to negotiate. We showed that tension exists for POs who must navigate two opposing versions of the same incident, which they described as bothersome, especially when they feel the versions are difficult to reconcile, specifically the variations between the “legal truth” with their client’s account of their criminal history. For POs, negotiating meaning between the official versions of events as deemed by the judge and their client’s, sometimes distorted, perception of their crimes, constitutes an emotionally taxing aspect of their job. While creating internal tension, we argue POs’ self-reflection and negotiation of PCSCs’ narratives of past, present, and future is an important requirement for treating PCSCs as citizens worthy of help and intervention, so they are understood by their POs, and thus collaboratively able to work toward creating safety and meaningful reintegration (Duff, 2021). Albeit this takes significant time and energy on the part of the PO, we argue it is critical for POs to take the time to seek to understand, make sense of, and work with their clients’ narratives to initiate meaningful change and accountability. This positions POs as change agents, rather than just case managers, by planning, prioritizing, and effectively achieving change with their clients (see Bourgon et al., 2012; Lovins et al., 2018). Tension also exists insofar as POs must make sense of and negotiate conflicting emotions regarding their own attitudes and feelings toward PCSCs clients. On the one hand, POs described PCSCs as generally pleasant and easy to manage, and yet, knowledge about their clients’ criminal history and details about their crime made difficult for them to accept these more positive feelings toward their clients. In other words, POs struggled to separate clients’ past behavior with how they experienced PCSCs clients as penal subjects. While our data show how this creates internal tension for the PO, we also argue that separation of the person from their past behavior may serve a productive function, insofar as the separation creates nuance and reduces generalizability of PCSCs. POs described PCSCs as pleasant to deal with, even friendly and kind, but with cognitions and beliefs that require intervention and that must be addressed.

POs’ narratives revealed that while PCSCs may be easier to supervise, this may be partly due to employing a simplistic label (i.e., “sex offender”) that depicts generically a monolithic type of discredited outsider (Becker, 1963). This is problematic in that it does not lend well to understanding the nuance that is required for addressing public safety and rehabilitative needs around the supervision of PCSCs. For example, in addition to negotiating understanding between the PCSC’s past criminality and sexual inclinations and their conduct as a penal subject, POs also voiced there is a need for understanding PCSCs clients on a continuum of harm (i.e., rapist vs. near-age-of-consent historical offender). In addition, POs develop understanding of various facets of a person’s past and current behavior, which affect POs’ ability and feelings around supervision.

To navigate different feelings and create nuance, POs describe their capacity as flexible, to “go with the flow” in separating wrongdoing from the human being, and trying to connect with clients as best as possible. As our data reveal, for POs, this includes maintaining a non-judgmental attitude, trying to help clients overcome shame and embarrassment that impede POs’ ability to offer help, and navigate external constraints, such as lack of programming and resources that are needed for proper assessment and intervention.

Our data suggest that for POs, tension in supervising PCSCs is related to imagining, negotiating, and working through a balance between what POs think are the best and worst case scenarios when supervising PCSCs clients. POs imagine one of the “worst case” scenarios to be a PCSC client who is paroled (a decision that is ultimately out of the hands of the PO) but has failed to accept any responsibility and accountability for their crimes. On the other side, a “best case” scenario refers to a good, open, and productive relationship between POs and PCSC clients, and the ability of the PO to help their client move toward desistance and change in behavior and thinking. Such productive skills and strategies aimed toward rehabilitation have been shown to be necessary to a reduction in recidivism (Chadwick et al., 2015; Labrecque et al., 2022) Necessary here for PCSCs is to accept responsibility and build social capital, “making good to offenders by enabling them to achieve inclusion and participation in society” through progressive and positive reframing of their identities (McNeill, 2006, p. 57).

**Figure 1.**
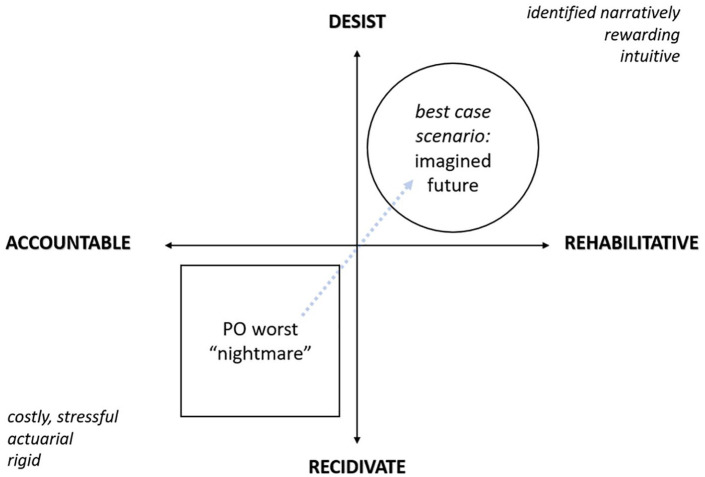
A four-point axis of parole work tension.

Looking to Figure 1, the “best case” scenario focuses on conceptions of the PCSCs’ future and on working toward creative long-lasting change (see also Maier, 2021). In other words, an orientation to “best case” scenario is an aspirational shift about an imagined future (Watkins et al., 2011, p. 282)—post release from prison—which is favorable to society and achieved through meaningful dialog between POs and their clientele (Duff, 2003, 2021).

However, POs who focus purely on risk (e.g., to the PCSC and publicity) are problematic, especially in institutional settings, inherently located in the lower-left quadrant of our four-point axis of tension where accountability/recidivism is prioritized and likeliest—the exact opposite outcome we want to promote for PCSCs in re-entry. While POs describe being stressed, related to spiraling incivility in the carceral workplace culture (Andersson & Pearson, 2013), this creates a tension in outcomes. Where POs attend workplaces where they feel strained, so too do PCSCs experience that stress during interactions with such a system (for more on offense analog and offense reduction behaviors, see Gordon & Wong, 2015). This is a missed opportunity building the therapeutic alliance necessary for working collaboratively toward desistance (Ricciardelli, 2018). We reiterate that a best-case scenario is therefore an outcome of rehabilitative-desistance as illustrated by the upper right quadrant in Figure 1.

As POs describe the need to be flexible in their own thinking and supervision, the same is true in therapy for PCSCs (i.e., programming) which needs to be flexible, accessible, and relevant. POs described some PCSCs getting little or no treatment, especially when they are assessed as relatively low risk, for example an incest cases where the risk factors can be managed (i.e., by removing the PCSCs’ own children from the situation), or when they lived in more remote or rural areas where treatment services were scarce. Thus, on a practical level, examining what sorts of programs are needed for whom is critical for ensuring PCSCs have access to relevant and helpful programming. Meaningful access to therapy (not one size fits all “programming”) stands in stark contrast with how punitiveness remains in tension with civic responsibility to help others in the pursuit of rehabilitation. Meaningful access to relevant programming is critical for both the PCSC as well as the PO’s ability to perform their job. As interviews reveal, some POs were concerned about the lack of available resources, or they desired greater shared responsibility between different treatment providers when it comes to PCSC assessment and intervention. Thus, in addition to creating accessible and meaningful programs, important remains to create and strengthen inter-agency collaborations that set up PCSCs with multiple access points to support (Mcgrath et al., 2002).

## Limitations and Future Research

Although sex offenses and PCSCs were not the focal point of our research, interview participants regularly brought up the topic. This suggests the supervision of PCSC clients is a valued topic to POs and thus warrants further academic attention to better understand POs’ feelings and experiences. Further research could investigate how geographical location and access to resources further impacts POs’ perceived ability to work with PCSCs, as well as how POs navigate external constraints to their work (e.g., lack of resources).

Furthermore, research that brings into conversation POs’ experiences with PCSCs’ perceptions and experiences around their own supervision would augment parole research, and aid in creating necessary programs and interventions for PCSCs. For example, our data show that POs believe it is shame and embarrassment that lead some PCSCs to not open up and seek support. Our study, however, only captures POs’ beliefs and points of view. Interviews with PCSC clients on parole could provide further insight and nuance into PCSCs’ feelings around supervision and toward their PO, including what PCSCs themselves believe are the barriers to engaging with their PO and other services. Research focused on PCSC experiences could help POs and other actors design programs and supervision styles targeted toward engaging with PCSCs clients specifically.

## Conclusion

Drawing on a sample of 150 POs in Canada, in the current article, we examined how frontline POs perceive, feel about, and seek to work with PCSCs clients on their caseload. Our data suggest the supervision of PCSCs is imbued with *tension.* Tension refers to both POs’ need for internal reflection and the negotiation of complex feelings that surround supervising a discredited group of people, while seeking to act as a professional rehabilitative and supportive figure for all clients. Tension also results from various external factors, such as the lack of available programming and shared responsibility between POs and other professionals, which puts added stress and responsibility on the PO to act as the central figure in the management of PCSCs. Thus, beyond drawing attention to how POs conceive of PCSCs, our data also draws attention to some particular facets of occupational stress experienced by POs. We show that attention to POs’ occupational experiences with particular client groups reveals much needed empirical nuance into POs’ feelings and experiences around their work, thereby augmenting and extending existing research on the realities and challenges of parole work.

## Notes

1. We acknowledge the “big five” personality traits (i.e., openness to experience, conscientiousness, extraversion, agreeableness, and neuroticism) are also worth exploring for POs but remain interested in situational factors currently (Sur & Ng, 2014).
